# Magnetic resonance imaging detected radiation-induced changes in patients with proton radiation-treated arteriovenous malformations

**DOI:** 10.1177/20584601211050886

**Published:** 2021-11-01

**Authors:** Maria Correia de Verdier, Elisabeth Ronne-Engström, Ljubisa Borota, Kristina Nilsson, Erik Blomquist, Johan Wikström

**Affiliations:** 1 Department of Surgical Sciences, Section of Neuroradiology, 59592 Uppsala University, Uppsala, Sweden; 2 Department of Neuroscience, Section of Neurosurgery, 59592 Uppsala University, Uppsala, Sweden; 3 Department of Immunology, Genetics and Pathology, Section of Oncology, 59592 Uppsala University, Uppsala, Sweden

**Keywords:** Magnetic resonance imaging, proton therapy, intracranial arteriovenous malformations

## Abstract

**Background:**

Treatment of intracranial arteriovenous malformations (AVMs) includes surgery, radiation therapy, endovascular occlusion, or a combination. Proton radiation therapy enables very focused radiation, minimizing dose to the surrounding brain.

**Purpose:**

To evaluate the presence of radiation-induced changes on post-treatment MRI in patients with AVMs treated with proton radiation and to compare these with development of symptoms and nidus obliteration.

**Material and Methods:**

Retrospective review of pre- and post-treatment digital subtraction angiography and MRI and medical records in 30 patients with AVMs treated with proton radiation. Patients were treated with two or five fractions; total radiation dose was 20–35 physical Gy. Vasogenic edema (minimal, perinidal, or severe), contrast enhancement (minimal or annular), cavitation and nidus obliteration (total, partial, or none) were assessed.

**Results:**

26 of 30 patients (87%) developed MRI changes. Vasogenic edema was seen in 25 of 30 (83%), abnormal contrast enhancement in 18 of 26 (69%) and cavitation in 5 of 30 (17%). Time from treatment to appearance of MRI changes varied between 5 and 25 months (median 7, mean 10). Seven patients developed new or deteriorating symptoms that required treatment with corticosteroids; all these patients had extensive MRI changes (severe vasogenic edema and annular contrast enhancement). Not all patients with extensive MRI changes developed symptoms. We found no relation between MRI changes and nidus obliteration.

**Conclusion:**

Radiation-induced MRI changes are seen in a majority of patients after proton radiation treatment of AVMs. Extensive MRI changes are associated with new or deteriorating symptoms.

## Introduction

Intracranial arteriovenous malformations (AVMs) are believed to be developmental, with an annual detection rate of 1 in 100, 000.^1–3^ Patients are at increased risk for intracranial hemorrhage and neurological symptoms, but may also be asymptomatic. AVMs can be treated with surgery, radiation, endovascular occlusion or a combination of these methods. The decision of treatment method has to consider many factors, such as the patient’s age, neurological status, associated clinical risk factors, angioarchitectural features of the AVM and also the patient’s choice.^4^ Radiation therapy is non-invasive. After radiation therapy, there is however a latency period before treatment effect and as long as there is still a residual nidus there is a remaining risk for hemorrhage. The patients are also at risk for radiation-induced complications, both early (e.g., edema in the adjacent brain) and late (e.g., leukoencephalopathy, necrosis, cysts, and tumors).^5–11^ Radiation therapy can be administered with photons or heavy charged particles (e.g. protons in this case). Photon radiation therapy is widely used and the occurrence of treatment related MRI findings has been studied in several previous works.^12^ Proton radiation therapy has the advantage of enabling very focused radiation, giving a homogenous dose to the target while minimizing dose to the surrounding brain parenchyma. Proton treatment facilities treating AVMs are however scarce and there is limited data on normal MRI findings after treatment.^13,14^ There are previous studies investigating the appearance of vasogenic edema after radiation therapy as a possible predictor for nidus obliteration.^14–21^

The purpose of the present study was to evaluate the emergence of radiation-induced changes on post-treatment MRI in patients with AVMs treated with proton radiation therapy. We aimed further to assess the possible relation between post-treatment MRI findings and development of clinical symptoms and nidus obliteration.

## Material and methods

At our institution, all AVMs are reviewed at a multidisciplinary neurovascular conference and specialists who perform surgery, endovascular treatment and radiotherapy decide in consensus the safest and most effective treatment for each patient. Patients with AVMs treated with proton radiation therapy between 2002 and 2015 and examined with pre-treatment digital subtraction angiography (DSA) and MRI and post-treatment MRI were eligible for inclusion in the study. A total of 30 patients were included. The routine follow-up protocol after proton radiation therapy during this time period included MRI after 6, 12, 24 and 36 months followed by DSA if there was no visible AVM nidus left, otherwise follow-up with yearly MRI continued. A retrospective review of the radiological images (pre-treatment DSA and MRI and all available post-treatment DSA and MRI), patients’ medical records and treatment protocols was performed in March 2021. Institutional Review Board approval was obtained and guidelines on patient consent have been met.

### Clinical information

The patients’ medical records and treatment protocols from Uppsala University Hospital were retrospectively reviewed. All the patients were treated at The Svedberg Laboratory in Uppsala between 2002 and 2015. The treatment method has been previously described in detail.^14^ The normal fractionation was two fractions, the most common dose 12 physical Gy × 2, but was decreased in some cases due to critical structures close by. In three patients, with larger nidus, the fractionation scheme was altered to five fractions with lower dose per fraction. All patients received treatment with corticosteroids in connection with the radiation treatment. Presence of new or deteriorating neurological symptoms that required additional steroid treatment was noted. Two patients received additional treatment of the AVM. One patient received radiotherapy with photons (gamma knife) 50 months after proton treatment when follow-up DSA showed a small remaining nidus. The other patient received embolization of an intranidal aneurysm and arteriovenous fistula 88 months after proton treatment when follow-up MRI showed a growing intranidal aneurysm. One year later the patient developed hemorrhage from the residual nidus and the remaining nidus was completely embolized.

### Digital subtraction angiography

Pre-treatment DSA images were assessed by a neurointerventionist (L.B.). We assessed nidus size, AVM location, number of feeders, number and location of draining veins, presence of and type of aneurysm and we calculated Spetzler–Martin score.^22^ All available post-treatment DSA images were assessed by a neurointerventionist (L.B.) for evaluation of obliteration of the AVM nidus and graded as total (entire nidus obliterated), partial (<100% obliteration), or none (no change in size of nidus).

### Magnetic resonance imaging

**Figure 1.**
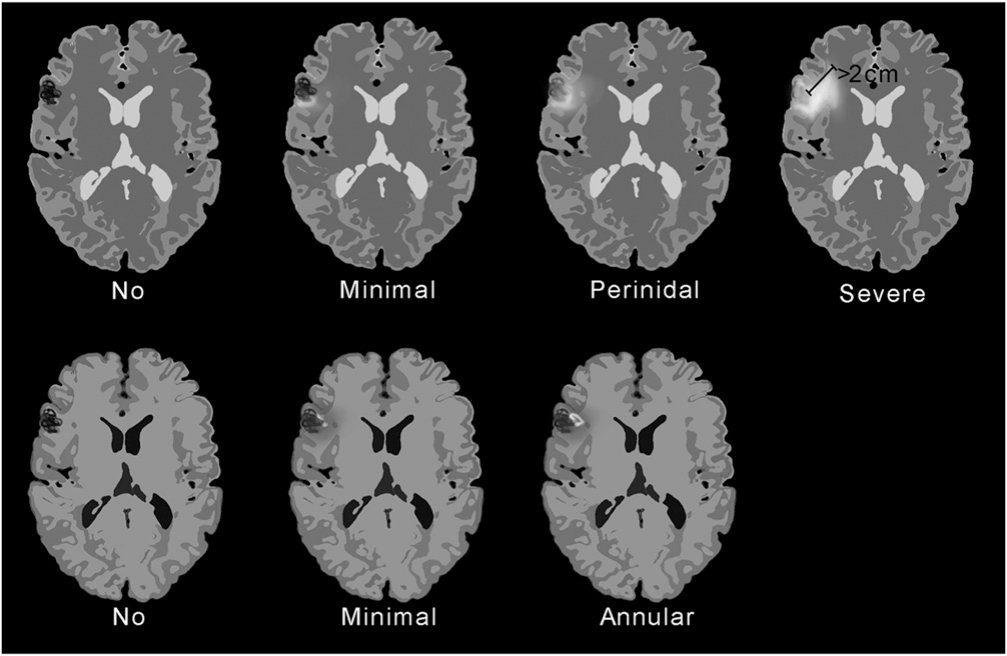
Classification of radiation-induced MRI findings after proton-treated AVMs used in this article. MRI: Magnetic resonance imaging; AVMs: Arteriovenous malformations.

All MRI images were separately assessed by two radiologists (J.W. and M.C.V.), blinded to the patients’ symptoms. The presence of vasogenic edema, contrast enhancement and cavitation was assessed on post-treatment MRI, with pre-treatment MRI for comparison. When the two radiologists disagreed, a consensus reading was conducted. Two patients received additional treatment of the AVM and their MRI after the additional treatments were excluded. The radiological classification used in this article is shown in Figure 1. We used a modification of previously published classifications.^21,23^ Vasogenic edema was defined as white matter hyperintensity on images from T2-weighted and/or fluid-attenuated inversion recovery (FLAIR) sequences, that was not seen on pre-treatment MRI and classified as no edema, minimal edema (traces or incomplete rim), perinidal (complete rim in the white matter surrounding the nidus or previous nidus location), or severe (as perinidal but the rim at some location at least 2 cm thick measured on axial images). Contrast enhancement was defined as extravascular hyperintense signal on images from contrast enhanced T1-weighted sequence and classified as no enhancement, minimal enhancement (traces or incomplete ring), annular enhancement (complete ring) and not gradable (when there was no intravenous contrast agent administered). Cavitation was defined as a lesion with fluid signal on images from T1- and T2-weighted sequences. All post-treatment MRI were assessed for evaluation of obliteration of the AVM nidus and graded as total (entire nidus obliterated), partial (<100% obliteration) or none (no change in size of nidus).

### Statistical analysis

Statistical analysis was performed using IBM SPSS Statistics 24 (IBM SPSS, Inc.). Inter-rater reliability to determine the consistency among raters for the classification of findings on post-treatment MRI was calculated using linear weighted Kappa. A value of 0.21–0.40 was considered fair agreement; 0.41–0.60, moderate agreement; 0.61–0.80, substantial agreement; 0.81–1.00, almost perfect. Kaplan–Meier survival curves were used to illustrate the appearance of radiation-induced changes over time. In cases where more extensive changes were observed without prior observation of less extensive changes, we chose to regard the appearance of more extensive changes also as appearance of less extensive changes for the analysis. Pearson’s Chi-square test and Fischer’s exact test were used to determine relationship between groups (post-treatment MRI findings and development of clinical symptoms or nidus obliteration). For Fisher’s exact test, MRI findings were regrouped into categories; vasogenic edema was regrouped into “no or minimal” and “perinidal or severe,” and contrast enhancement was regrouped into “no or minimal” and “annular”. Mann–Whitney *U* test was used to determine relationship between time to development of radiological findings and degree of nidus obliteration. *p*<0.05 was considered to be statistically significant.

## Results

### Clinical information

Thirty patients were included in the study; patient demographics, AVM characteristics and treatment parameters are described in Supplementary Table 1 and outcome variables are described in Supplementary Table 2. Time between treatment and review of medical records was between 72 and 226 months (median 156, mean 145). Seven patients developed new or deteriorating symptoms that were associated with radiation therapy and required treatment with corticosteroids (e.g. headache, speech disturbance, nausea, more frequent seizures and worsening of paresis). Time from radiation treatment to start of treatment with corticosteroids was between 4 and 23 months (median 9, mean 11). Two additional patients reported symptoms after radiation treatment, but the association with radiation therapy was unclear and they were hence not included in the symptomatic group in the analysis. One patient developed a hemorrhage from the residual nidus 8 years after radiation therapy.

### Radiological follow-up

Some patients did not adhere to the routine radiological follow-up protocol for various reasons (including resettlement, reluctance to DSA or MRI, symptoms requiring repeated or more frequent MRI, and death due to other causes). Twenty-two patients had undergone post-treatment DSA until March 2021. DSA could confirm decrease of nidus size in all 22 patients; total obliteration in 14 and partial obliteration in 8. Eight patients did not undergo DSA and obliteration is based on their latest MRI. A decrease in nidus size on MRI was observed in seven of the eight patients; total obliteration in two and partial obliteration in 5. One patient did not undergo DSA and nidus was not seen on pre-treatment MRI.

### Magnetic resonance imaging findings

**Figure 2.**
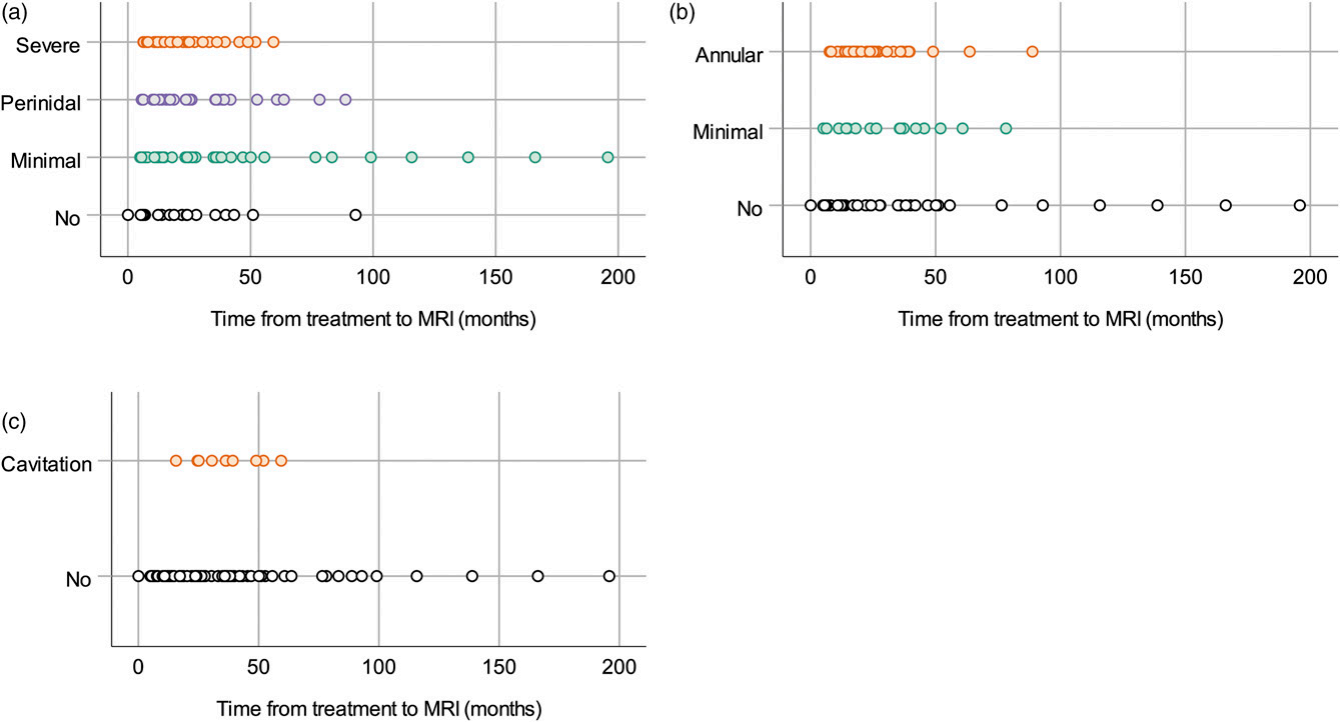
Presence of radiation-induced MRI findings in all available post-treatment MRI in 30 patients with proton-treated AVM. Vasogenic edema of different extent (no, minimal, perinidal, or severe) in 129 post-treatment MRI (2a), contrast enhancement of different extent (no, minimal, or annular) in 105 post-treatment MRI (2b), and presence of cavitation in 129 post-treatment MRI (2c). MRI: Magnetic resonance imaging; AVMs: Arteriovenous malformations.

**Figure 3.**
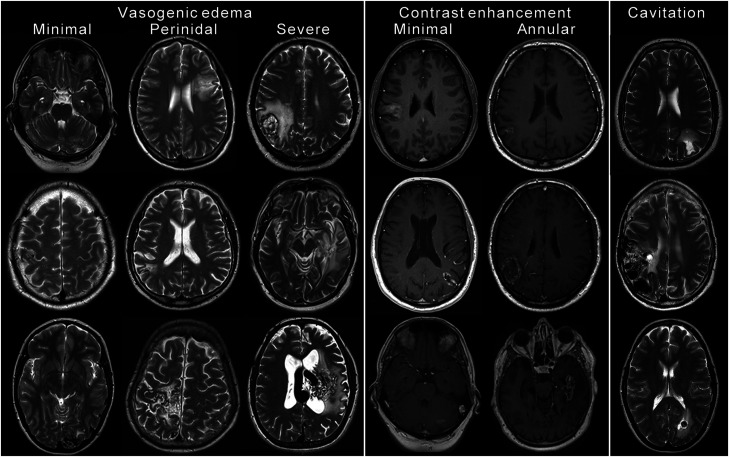
Example images from different patients demonstrating radiation-induced MRI findings after proton-treated AVMs according to our classification; vasogenic edema (minimal, perinidal, or severe), contrast enhancement (minimal or annular), and cavitation. MRI: Magnetic resonance imaging; AVMs: Arteriovenous malformations.

**Figure 4.**
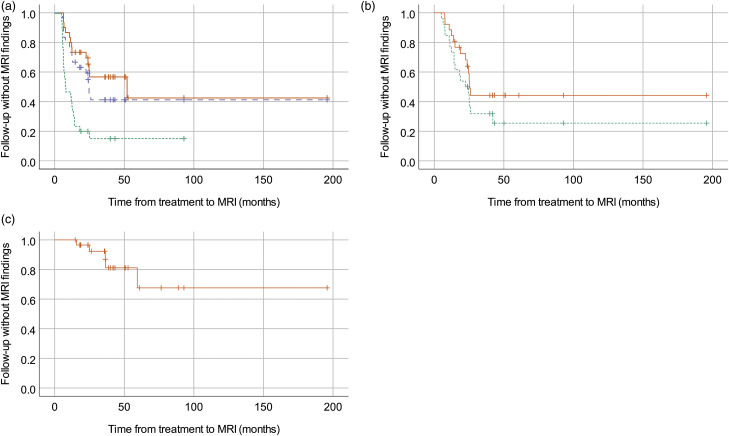
Kaplan–Meier survival curves for the appearance of vasogenic edema (4a, n 30), contrast enhancement (4b, n 26), and cavitation (4c, n 30) on post-treatment MRI. Vasogenic edema of different extent (minimal = green dotted line, perinidal = purple dashed line, or severe = orange continuous line), contrast enhancement (minimal = green dotted line or annular = orange continuous line), and cavitation (presence of cavitation = orange continuous line) is distinguished. The curves represent fraction of patients without new MRI findings. Patients lost to follow-up are indicated on the curve as tick marks. In cases where more extensive changes were observed without prior observation of less extensive changes, we chose to regard the appearance of more extensive changes also as appearance of less extensive changes for the analysis. MRI: Magnetic resonance imaging.

A total of 129 post-treatment MRI were included in the study. Of these, 105 were performed with contrast enhanced T1-weighted images. The MRI was done between 0 (1 day) and 196 months after treatment (median 23, mean 29). Each patient was examined with between 1 and 12 MRI (median 4, mean 4). Median length of follow-up from treatment with MRI was 40 months (range 15–196, mean 49). Inter-rater reliability for the raters of the post-treatment MRI was 0.71 for vasogenic edema, 0.69 for contrast enhancement and 0.51 for cavitation. MRI findings (vasogenic edema, contrast enhancement and/or cavitation) were seen in 26 of 30 patients (87%). The frequencies of different MRI findings are shown in Table 1 and Figure 2. Examples of MRI findings from different patients are shown in Figure 3. Time from treatment to first appearance of any radiological finding (vasogenic edema, contrast enhancement, or cavitation) varied between 5 and 25 months (median 7, mean 10). Time from treatment to first appearance of vasogenic edema was between 5 and 25 months (median 7, mean 9). Contrast enhancement first appeared between 5 and 42 months (median 15, mean 17). Time from treatment to appearance of cavitation was between 16 and 59 months (median 36, mean 35). Kaplan–Meier survival curves that illustrate the interval between treatment and MRI findings are shown in Figure 4.

**Table 1.** Frequencies of radiological changes in patients on post-treatment magnetic resonance imaging.

	Vasogenic edema (n 30)		Contrast enhancement (n 26^1^)		Cavitation (n 30)
Yes	25 (83%)	Yes	18 (69%)	Yes	5 (17%)
Minimal	9 (30%)	Minimal	5 (19%)		
Perinidal	3 (10%)	Annular	13 (50%)		
Severe	13 (43%)				
No	5 (17%)	No	8 (31%)	No	25 (83%)

^1^Intravenous contrast agent was not administered to four patients.

### Relation between post-treatment MRI findings and symptoms

We found a significant relation between MRI findings (both vasogenic edema and contrast enhancement) and development of symptoms (*p* < 0.05, Table 2). We did not find any relation between cavitation and symptoms. All seven patients with symptoms that required treatment with corticosteroids had extensive radiological findings (combination of severe vasogenic edema and annular contrast enhancement). One patient with symptoms and extensive radiological findings was recommended corticosteroids, but there is no follow-up information. Two patients with extensive radiological findings (combination of severe vasogenic edema and annular contrast enhancement) did not develop symptoms.

**Table 2.** Relation between post-treatment magnetic resonance imaging findings and new or deterioration symptoms.

	Vasogenic edema (n 28^1^), *p* < 0.05				Total
	No	Minimal	Perinidal	Severe	
No treatment	5	8	3	5	21
Treatment with corticosteroids	0	0	0	7	7
Total	5	8	3	12	28

	Contrast enhancement (n 24^1,2^), *p* < 0.05			Total
	No	Minimal	Annular	
No treatment	8	4	5	17
Treatment with corticosteroids	0	0	7	7
Total	8	4	12	24

	Cavitation (n 28^1^), *p* > 0.05		Total
	No	Yes	
No treatment	18	3	21
Treatment with corticosteroids	6	1	7
Total	24	4	28

Pearson’s Chi-square test and Fischer’s exact test were used to determine relationship between groups. Not all assumptions for Pearson’s Chi-square test were met (cells with expected count less than five). For Fisher’s exact test, MRI findings were regrouped into categories; vasogenic edema was regrouped into “no or minimal” and “perinidal or severe,” and contrast enhancement was regrouped into “no or minimal” and “annular.”

^1^Two patients were excluded from the analysis; one patient with severe vasogenic edema and annular contrast enhancement developed symptoms and was recommended treatment but there are no further follow-up records and one patient with minimal vasogenic edema and minimal contrast enhancement developed symptoms and received treatment with corticosteroids but the symptoms were believed not to be related to the radiation treatment.

^2^Intravenous contrast agent was not administered to four patients.

### Relation between post-treatment MRI findings and obliteration

We found neither any relation between MRI findings and obliteration of nidus nor between time to development of MRI findings and obliteration of nidus.

## Discussion

In this study, we examined 30 patients who have been treated with proton radiation therapy for intracranial AVMs. We found a high frequency (87%) of radiation-induced changes on MRI (vasogenic edema, contrast enhancement and/or cavitation). We found a relation between MRI findings and symptoms but we did not see any relation between MRI findings and nidus obliteration.

Diagnosing radiation-induced changes on MRI is complicated due to lack of studies correlating MRI findings with histopathological information. Histopathological studies on AVMs after radiosurgery show damage to endothelial cells, followed by progressive thickening of the intimal layer caused by proliferation of smooth-muscle cells and finally, cellular degeneration and hyaline transformation.^10,24^ Both vasogenic edema and contrast enhancement may be a manifestation of desired treatment response, radiation-induced modifications and complications. Another difficulty is that, there are several scoring systems that have been proposed for evaluation of post-treatment MRI,^19,21,23,25,26^ and there is no consensus on what scoring system to use. We chose a modified version of a previously published visual grading scoring system.^21,23^ A more stringent definition of extensive MRI findings not depending on the nidus size renders the classification easier to use and more consistent. No previous study has evaluated the inter-rater agreement of their scoring systems; the agreement in our study was moderate to substantial.

The presence of edema was observed in more patients (83%) than in previous studies on proton treated AVMs, but we used different definitions of edema.^13,14^ In studies on patients treated with photon radiation, the range of reported edema is between 4 and 100%, which might reflect differences in study design (e.g., different definitions of edema, radiation doses, patient population, lesions included, follow-up times etc.).^12^ Early after proton treatment, edema surrounding the nidus due to the increased capillary permeability caused by endothelial and subendothelial damage may be present. The progressive proliferation of intimal muscle cells and narrowing of the vessel lumina seen months to years after treatment may cause edema due to venous congestion or ischemia. Delayed radiation-induced complications, or complications caused by a residual nidus, may also cause MRI findings surrounding the nidus.

We observed contrast enhancement in 69% of the patients. We are not aware of any previous study reporting the frequency of contrast enhancement after proton radiation therapy of AVMs. There is a large range of reported frequencies of contrast enhancement after photon therapy; between 16% and 66%.^17,20,21,23,27^ The damage to endothelial cells seen early after proton radiation treatment of AVMs may lead to a disruption of the blood–brain barrier causing contrast enhancement in the adjacent brain parenchyma. Contrast enhancement may also been seen as a result of complications after radiation therapy (necrosis or tumors) or as a complication of a residual nidus (ischemia or hemorrhage).

Five patients (17%) developed cavitation. It should be noted that this may be a heterogeneous group; including both radiation-induced necrosis, cyst formations and other types of new developed fluid collections. In previous studies on both proton and photon radiation therapy, cyst formation has been observed in 2% of patients, usually appearing several years after treatment.^7,11^ The reported frequencies of radiation necrosis after photon radiation therapy vary between 0 and 13%.^19–21,23^

We found that time from treatment to first appearance of radiation-induced image changes (vasogenic edema, contrast enhancement, or cavitation) was between 5 and 25 months (median 7 months). To our knowledge, there is no other study reporting the time between proton radiation treatment and MRI findings. In concordance with previous studies on photon-treated AVMs, we found that there can be a long interval between treatment and development of MRI changes.^19,23,28^ We believe that this is caused by the broad spectrum of histological etiologies providing MRI findings with similar appearance.

In our study, seven (25%) patients developed symptoms that required treatment with corticosteroids and they all had extensive radiation-induced findings on post-treatment MRI (severe vasogenic edema and annular contrast enhancement). Not all patients with extensive MRI findings did develop symptoms though. There is only one previous study investigating the relation between radiation-induced radiological findings after proton treatment and development of new or deteriorating neurological symptoms, in which symptomatic edema was found in 11 of 65 patients (17%).^14^ This study has an overlap with our study in inclusion of patients and 16 of the patients in our study have been described before. There are previous studies on photon treated AVMs (linear accelerator (LINAC) and gamma knife) reporting a correlation between more extensive MRI findings and development of symptoms.^19,20,23^

It would be desirable if radiological findings could predict treatment result, primarily because of the latency period before complete treatment effect and remaining bleeding risk from residual nidus. We could not find any relation between type, extent or time to appearance of MRI changes and nidus obliteration. Blomquist et al. found that patients with edema on post-treatment CT or MRI had higher rate of total nidus obliteration.^14^ Studies on photon radiation therapy (gamma knife and LINAC) report MRI changes to be a positive predictive factor for nidus obliteration.^15–17^ In a meta-analysis on photon-treated AVMs, no relation was seen between edema and nidus obliteration.^12^ Some authors report a correlation between more extensive MRI changes after gamma knife or LINAC and nidus obliteration.^18,19,26^ Others suggest that more extensive MRI changes could predict earlier nidus obliteration.^20,26^ Difference in study designs, no standardized scoring system for radiation-induced MRI changes and different modalities used to evaluate response is a probable explanation for the difference in results in these studies.

Various limitations of the study should be mentioned. The retrospective study design entails that we partly had to rely on others for data collection and accurate record keeping. The investigated patient population is small and heterogeneous with a variable length of follow-up time. Not all patients followed the routine follow-up protocol and not all patients underwent post-treatment DSA for assessment of nidus obliteration.

In conclusion, radiation-induced MRI changes were seen in a majority of AVM patients after proton radiation treatment. Patients with symptoms that required treatment with corticosteroids had extensive MRI changes but not all patients with extensive MRI changes had symptoms that required treatment. MRI changes were not found to be predictive for nidus obliteration.

## Supplemental Material

sj-pdf-1-arr-10.1177_20584601211050886 – Supplemental Material for Magnetic resonance imaging detected radiation-induced changes in patients with proton radiation-treated arteriovenous malformations Click here for additional data file. Supplemental Material, sj-pdf-1-arr-10.1177_20584601211050886 for Magnetic resonance imaging detected radiation-induced changes in patients with proton radiation-treated arteriovenous malformations by Maria Correia de Verdier, Elisabeth Ronne-Engström, Ljubisa Borota, Kristina Nilsson, Erik Blomquist and Johan Wikström in Acta Radiologica Open
