# Supplementary material for: Magnetic resonance imaging detected radiation-induced changes in patients with proton radiation-treated arteriovenous malformations
Source: Acta Radiol Open. 2021 Nov 1;10(10):20584601211050886. doi: 10.1177/20584601211050886 (PMC8649916; doi:10.1177/20584601211050886)
Supplement: sj-pdf-1-arr-10.1177_20584601211050886 – Supplemental Material for Magnetic resonance imaging detected radiation-induced changes in patients with proton radiation-treated arteriovenous malformations [file sj-pdf-1-arr-10.1177_20584601211050886.pdf]

Supplementary tables

Supplementary table 1. Patient demographics, AVM characteristics and treatment parameters

| | |
|---|---|
| Sex | |
| - Male | 19 (63%) |
| - Female | 11 (37%) |
| Age at treatment (years) | 12 – 65 (mean 44, median 46.5) |
| Symptoms at diagnosis | |
| - Asymptomatic | 6 (20%) |
| - Headache | 11 (37%) |
| - Neurological deficit | 11 (37%) |
| - Seizure | 10 (33%) |
| - Hemorrhage | 6 (20%) |
| - Tinnitus | 3 (10%) |
| Prior treatment of AVM | |
| - No | 26 (87%) |
| - Radiation therapy | 0 |
| - Embolization | 3 (10%)[1,2] |
| - Surgery | 1 (3%) |
| Size of nidus | |

| | |
|---|---|
| - <3 cm | 14 (47%) |
| - 3-6 cm | 10 (33%) |
| - >6 cm | 6 (20%) |
| Location | |
| - Cortical | 26 (87%) |
| - Subcortical | 25 (83%) |
| - Ventricular | 7 (23%) |
| - Corpus callosum | 1 (3%) |
| - Frontal | 8 (27%) |
| - Temporal | 7 (23%) |
| - Parietal | 13 (43%) |
| - Occipital | 8 (27%) |
| - Basal ganglia | 3 (10%) |
| - Internal capsule | 2 (7%) |
| - Cerebellar hemisphere | 3 (10%) |
| - Vermian | 1 (3%) |
| - Deep cerebellar nuclei | 1 (3%) |
| - Brain stem | 0 |
| Aneurysm | |

| | |
|---|---|
| - No | 25 (83%) |
| - Flow related | 4 (13%) |
| - Intranidal | 1 (3%) |
| Feeding arteries | |
| - 1 | 4 (13%) |
| - 2-3 | 8 (27%) |
| - > 3 | 18 (60%) |
| Venous drainage | |
| - Single draining vein | 8 (27%) |
| - Superficial drainage only | 18 (60%) |
| - Deep drainage only | 1 (3%) |
| - Both superficial and deep drainage | 11 (37%) |
| Spetzler-Martin score | |
| - 1 | 6 (20%) |
| - 2 | 10 (33%) |
| - 3 | 6 (20%) |
| - 4 | 5 (17%) |
| - 5 | 3 (10%) |
| Target volume at radiation | <1 – 62 ccm (mean 10.3, median 6.5) |

| | |
|---|---|
| Number of fractions | |
| - 2 fractions | 27 (90%) |
| - 5 fractions | 3 (10%) |
| Radiation dose per fraction (physical Gy) | 7 – 12 mean (11.2, median 12.0) |
| - 7 Gy | 3 (10%) |
| - 10 Gy | 2 (7%) |
| - 11 Gy | 9 (30%) |
| - 12 Gy | 16 (53%) |
| Total radiation dose (physical Gy) | 20 – 35 (mean 24.2, median 24.0) |

1 – Target embolization for reduction of AVM nidus prior to proton radiation therapy

2 – Two patients underwent embolization for flow related aneurysms but the AVM nidus was not treated prior to proton radiation therapy

Supplementary table 2. Morbidity and outcomes

| | |
|---|---|
| Additional treatment of AVM | |
| - No | 28 (93%)[1] |
| - Radiation therapy | 1 (3%) |
| - Embolization | 1 (3%) |
| - Surgery | 0 |
| AVM-related hemorrhage after proton radiation therapy | |
| - No | 28 (97%) |
| - Yes | 1 (3%)[2] |
| Post-radiation symptoms requiring treatment with corticosteroids | 7 |
| Change in nidus based on DSA (n 22[3]) | |
| - No | 0 |
| - Partial obliteration | 8 |
| - Total obliteration | 14 |
| Change in nidus based on MRI (n 28[4]) | |
| - No | 0 |
| - Partial obliteration | 14 |
| - Total obliteration | 14 |
| Survival | |

| | |
|---|---|
| - Yes | 27 (90%) |
| - Death due to other causes | 3 (10%) |
| - Death due to AVM | 0 |

1 – One patient did undergo embolization for two flow related aneurysm but the AVM was not additional treated

2 – One patient developed hemorrhage from the residual nidus, the patient had received additional treatment and nidus had been partially embolized one year prior to the hemorrhage

3 – Twenty-two patients underwent post-treatment DSA

4 – In two patients nidus was not visible on pre-treatment MRI
